# Editorial: Research advances toward One Health in brucellosis

**DOI:** 10.3389/fmicb.2026.1815921

**Published:** 2026-03-09

**Authors:** Carlos A. Rossetti, Beatriz Arellano-Reynoso, Eduardo H. Gotuzzo, Angel A. Oñate, Claire Ponsart

**Affiliations:** 1 Instituto de Patobiología - Instituto de Patobiología Veterinaria (IP-IPVet), UEDD INTA-CONICET, Buenos Aires, Argentina; 2 Consejo Nacional de Investigaciones Científicas y Técnicas (CONICET), Buenos Aires, Argentina; 3 Facultad de Medicina Veterinaria y Zootecnia, Departamento de Microbiología e Inmunología, Universidad Nacional Autónoma de México, Mexico City, Mexico; 4 Instituto de Medicina Tropical Alexander von Humboldt, Universidad Peruana Cayetano Heredia, Lima, Peru; 5 Laboratorio de Inmunología Molecular, Facultad de Ciencias Biológicas, Departamento de Microbiología, Universidad de Concepción, Concepción, Chile; 6 Ministère de l'Agriculture et de la Souveraineté alimentaire, Marcy-l'Étoile, France

**Keywords:** biomarkers, *Brucella*, diagnosis, osteoarthritis, surveillance, vaccine

Although the concept of “One Health” was used in the past, it was formally formulated at the beginning of this Century to indicate that the health of humans, animals and plants are closely linked and interdependent with each other as well as with their shared environment.^1^ The concept also emphasizes that collaboration across disciplines contributes to addressing health challenges such as the prevention, detection, control and elimination of infectious diseases, increasing global health security.

Brucellosis is a neglected zoonotic infectious disease in most developing countries (Franc et al., 2018). It causes chronic illness with a variety of non-specific clinical signs in humans (fever, malaise, fatigue and arthritis) and abortion, infertility and decreased milk production in animals. These clinical manifestations cause significant economic losses in livestock and pose a risk to public health. It is estimated that hundreds of thousands of new human brucellosis cases appear each year (Moreno et al., 2022), while the livestock industry loses millions of dollars annually (Mendes et al., 2025).

Prevention measures and diagnostic tests are the best tools for preventing and controlling brucellosis, with vaccines and serological tests being the prototypes of these measures, respectively. However, despite a large amount of research in these topics, brucellosis vaccines are far from ideal, and no serological test by itself can identify a positive sample with enough specificity. Given the importance of improving these tools in creating a safe environment, these subjects are primarily addressed in our Research Topic.

As mentioned earlier, no vaccine for humans is licensed and veterinary vaccines present several shortcomings. There are four brucellosis veterinary vaccines available: *B. abortus* S19, *B. abortus* RB51, *B. melitensis* Rev.1 and *B. suis* S2. All of them are live attenuated strains and have several important drawbacks, including residual virulence, abortion risk, serodiagnostic interference and potential zoonotic transmission. Zoonotic transmission could occur through accidental inoculation or contact with infected material, including ingestion of unpasteurized infected dairy products from livestock with persistent shedding. Here, Boggiatto et al. characterized cellular and humoral immune responses to RB51, a commercially available rough *Brucella* vaccine strain against bovine brucellosis, in persistently colonized cattle. The authors demonstrated that cattle with delayed clearance of the RB51vaccine strain present a lack of peripheral anti-RB51 CD4+ T cell responses and a concurrently high humoral response of IgM and IgG anti-RB51. Even with limitations (only 1 excretor), this study provides an initial assessment of immune responses in an adult cow persistently colonized by the live attenuated vaccine, which will undoubtedly help to unravel the immune mechanisms that result in the delayed elimination of vaccine strains and allow for the development of safer brucellosis vaccines that would provide public health benefits.

Although it has been known for many years that vaccination campaigns are one of the main tools for preventing and controlling bovine brucellosis, this concept does not seem to be uniformly known among official actors, as reported by the work of Paixão et al. The authors conducted a situational analysis of brucellosis vaccination in cattle and buffaloes raised in Maranhão State (Brazil), and the study's conclusions are applicable worldwide, where animal brucellosis remains prevalent. Throughout a questionnaire, different obstacles to vaccination operations were identified, such as general knowledge about vaccination or updating on the National Program for Control and Eradication of Brucellosis Technical Regulations, and low adherence to wearing personal protective equipment. Other obstacles to vaccination identified by the authors were the poor quality of access roads to the properties and breeders with a limited number of bred calves in the vaccination age (3–8 months old). These factors hinder adherence to vaccination programs and emphasize the importance of adopting an integrated approach and cooperation between the public health and agricultural sectors.

Epidemiological surveillance is a useful tool for providing evidence of disease prevalence in a defined geographic area in specific animal species or humans. In this Research Topic, Nabi et al. and Luo et al. reported on brucellosis in small ruminants in northern Algeria, and cases of human brucellosis in Xinjiang, China, respectively. In the first article, the phylogenetic analysis of one *B. melitensis* strain isolated from a sheep milk sample revealed a high genetic similarity to other *B. melitensis* strains of human origin from North Africa and “European” cases associated with travel; whereas the second article clearly stated that many brucellosis patients comprised middle-aged young men primarily engaged in agriculture. The results of both studies highlight the risk that animal brucellosis poses to public health and the importance of developing effective control strategies for susceptible animals to prevent outbreaks in humans.

Serological diagnostic tests are the most cost-effective, and widespread tools for the routine detection of brucellosis in livestock and public health, although their sensitivity and specificity vary depending on the animal species infected, the *Brucella* species and the epidemiological situation (OIE, 2018). In this Research Topic, six original articles showcase new or modified techniques aimed at more accurate diagnosis of *Brucella* infection in animals and humans. Wang et al. developed and tested a SNP-based droplet digital polymerase chain reaction (SNP-ddPCR) method in 50 patients to detect *Brucella suis* S2 vaccine strain infections from wild-type infections. This method can be used not only for laboratory diagnostic identification of personnel exposed to *Brucella*-infected animals but also for the detection of S2 vaccine strain infections in livestock, thus preventing economic losses from misidentification and unnecessary culling. In two other articles, Xie et al. and Wu et al. developed and evaluated a recombinant protein-based ELISA for the serological diagnosis of human brucellosis. According to the results reported by Xie et al., the rBP26-based competitive ELISA achieved 100% sensitivity and specificity in the detection of human brucellosis and significantly outperformed the cELISA-based LPS mAb. On the other hand, the seven selected type 4 secretion system (T4SS) proteins demonstrated >91% sensitivity and specificity, indicating good performance compared with traditional diagnostic tests. The advantage of these two ELISAs over current serological diagnostic tests, which are primarily based on lipopolysaccharide (LPS), is their ability to avoid cross-reactivity with other Gram-negative bacteria, thus improving their specificity.

Qi et al. tested the ability of three specific chicken yolk antibodies (IgY) generated against a fusion protein containing the sequences of several *Brucella* outer membrane proteins, *Brucella* LPS, and whole-cell antigen for detecting *Brucella* compared to traditional IgG antibodies. IgY antibodies exhibited a higher detection limit for *B. abortus* compared to bovine IgG antibodies and no cross-reactivity with common foodborne pathogens. In addition, LPS-IgY and *Brucella*-IgY showed excellent detection capabilities in identifying *Brucella* in food samples. These results represent a promising alternative approach to detecting *Brucella* in food products, reducing the risk of transmission and ensuring public health and safety.

Biomarkers are biological molecules that can be measured and evaluated objectively and precisely to indicate a specific patient condition. In this Research Topic, the article by Li et al. used proteomics, bioinformatics and machine learning algorithms to identify diagnostic biomarkers in the serum of patients with brucellosis. After laboratory work and bioinformatics analysis, five proteins were selected. These five proteins, all of which are involved in the innate immune processes of brucellosis, demonstrated significant diagnostic value and may provide a valuable guide for identifying patients with clinical symptoms and concurrent risk factors but who have not yet developed antibodies against *Brucella*.

Canine brucellosis, caused by the rough and zoonotic species *B. canis*, faced serological diagnostic challenges including relatively low sensitivity and specificity of the tests. In addition, the commercially available serological test for *B. canis* diagnosis in the US became unavailable in 2022, leaving a gap in the availability of commercial tests for screening and diagnosis for canine brucellosis. With the goal of solving this problem, LeCuyer et al. evaluated the performance of three different methodologies (*B. ovis* ELISA, immunochromatographic lateral flow assay-ILF- and indirect fluorescent antibody assay-IFA-) currently available, as serological tests of *B. canis* for use in veterinary diagnostic laboratories or as a point-of-care test. After performing an interlaboratory comparison of the three *B. canis* serologic assays on a panel of 56 canine sera positive or negative for *B. canis*, each at five or six different veterinary diagnostic laboratories, the interrater reliability of all assays was good. However, due to the limited number of samples analyzed and the limitations of each test, it will be necessary to analyze the positive samples simultaneously with a more specific test.

Finally, the review of Chen et al. focuses on osteoarthritis, which is one of the most common complications not only in human brucellosis but also in canine and porcine brucellosis, caused by *B. canis* and *B. suis*, respectively. In this article, the authors highlight recent advances in clinical features, molecular pathogenic mechanisms, and animal models used to understand *Brucella* osteoarticular infections, underlying the complexity of the pathogenesis of *Brucella* osteoarticular infections and the central role of the inflammation as a contributing factor to *Brucella*-induced bone loss.

Brucellosis is a prime example of an infectious disease that requires a One Health strategy for its prevention, control and eradication. We expect that the articles included in this Research Topic will contribute to combating the disease.

## Footnotes

1
http://www.who.int/health-topics/one-health#tab=tab_1
